# Genetic variations in the PI3K-PTEN-AKT-mTOR pathway are associated with distant metastasis in nasopharyngeal carcinoma patients treated with intensity-modulated radiation therapy

**DOI:** 10.1038/srep37576

**Published:** 2016-11-23

**Authors:** Qiaojuan Guo, Tianzhu Lu, Yan Chen, Ying Su, Yuhong Zheng, Zeng Chen, Chao Chen, Shaojun Lin, Jianji Pan, Xianglin Yuan

**Affiliations:** 1 Shengli Clinical Medicine College, Fujian Medical University, Fuzhou, Fujian Province, 350014, China; 2 Department of Clinical Laboratory, Fujian Provincial Cancer Hospital, Fuzhou, Fujian Province, 350014, China; 3 Department of Radiation Biology Laboratory, Fujian Provincial Cancer Hospital, Fuzhou, Fujian Province, 350014, China; 4 Department of Radiation Oncology, Fujian Provincial Cancer Hospital, Fuzhou, Fujian Province, 350014, China; 5 Fujian Provincial Key Laboratory of Translational Cancer Medicine, Fuzhou, Fujian Province, 350014, China; 6 Department of Oncology, Tongji Hospital, Huazhong University of Science and Technology, Wuhan, Hubei Province, 430030, China

## Abstract

Distant metastasis is the primary failure pattern of nasopharyngeal carcinoma(NPC) in intensity-modulated radiation therapy(IMRT) era. This study was conducted to find the impact of genetic variations in the phosphatidylinositol 3-kinase(PI3K)/phosphatase and tensin homologue(PTEN)/v-akt murine thymoma viral oncogene homologue(AKT)/mammalian target of rapamycin(mTOR) pathway on the risk of distant metastasis in NPC. We genotyped 16 single-nucleotide polymorphisms(SNPs) in five core genes in this pathway from 496 patients treated by IMRT with or without chemotherapy. The relationships between genetic polymorphisms and distant progression were evaluated. We observed that two loci in the AKT1 gene(rs3803300 and rs2494738 alone or combined) were associated with prognosis, with patients carrying at least one variant allele had significantly reduced risk of distant failure, especially in N2-3 group. In addition, we found that genetic variation may had some joint effect with N classification in recursive-partitioning analysis(RPA) analysis, with which patients were stratified into four different risk subgroups (RPA model): RPA1(low risk), RPA2(moderate risk), RPA3(high risk) and RPA4(highest risk). Our findings suggested that genetic variations within the PI3K signaling pathway modulate the development and invasion of NPC patients. Further research is needed to replicate the study in other centers and races, and to unravel the functional significance of these polymorphisms.

Nasopharyngeal carcinoma (NPC) is an endemic disease in Southeast Asia and southern China^1^. The application of chemotherapy and intensity-modulated radiation therapy(IMRT) have significantly improved the treatment outcomes. Even with the best available treatment in modern practice, retrospective reports of patients treated with IMRT over the last decade have revealed that 15% to 30% will experience failure at distant sites^2^. Tumor-nodal-metastasis (TNM) system is critical in predicting prognosis and facilitating treatment planning. However, a significant heterogeneity in treatment outcomes is observed for patients within the same clinical stages and a subset of patients are considered to be at higher risk of tumor progression and distant metastasis. Thus, it would be of clinical interest to identify prognostic factors for distant metastasis or tumor progression that would enable physicians to identify subgroups of patients who may benefit from more aggressive individualized therapy.

The PI3K/PTEN/AKT/mTOR pathway, which consists of phosphoinositide 3-kinase (PI3K), phosphatase and tensin homolog (PTEN), v-akt murine thymoma viral oncogene homolog (AKT), and mammalian target of rapamycin (mTOR), has been implicated in the regulation of angiogenesis and metastasis – both important processes in cancer development and progression^3^,^4^. Several literatures have been reported that genetic variations in this pathway are associated with clinical outcomes, invasion property, drug resistance to chemotherapy and treatment complications, including head and neck squamous cell carcinoma, esophageal cancer, cervical cancer, gastric cancer, colorectal carcinoma, lung cancer and bladder cancer^5^,^6^,^7^,^8^,^9^,^10^,^11^,^12^,^13^,^14^,^15^,^16^,^17^. Although the involvement of this signaling pathway in the development and invasion of NPC have been addressed in many literatures^4^,^18^,^19^,^20^,^21^,^22^, the clinical significance of genetic variations in this pathway remains unclear in NPC. Herein, we performed this study, which enrolled 496 NPC patients treated by IMRT with or without chemotherapy, aimed to identify the potential associations between genetic variations in PI3K/PTEN/AKT/mTOR pathway and the occurrence of distant metastasis in patients with NPC.

## Materials and Methods

### Ethical statement

This retrospective study was conducted in compliance with the policy of Fujian Provincial Cancer Hospital to protect the private information of patients enrolled. All methods were performed in accordance with the relevant guidelines and regulations of Fujian Provincial Cancer Hospital and was approved by its ethical committee. All subjects and/or guardians received and signed informed consent.

### Patients’ characteristics

This study included 496 patients with histologically diagnosed non-metastatic NPC who were recruited between January 2012 and May 2013 at Fujian Provincial Cancer Hospital and had blood samples available for analysis. None had history of previous treatment or prior malignancy. All of them completed a pretreatment evaluation according to our institutional protocol^23^ and staged according to the 7th AJCC staging system. Peripheral blood specimens for genetic analysis were collected from each patient at the time of diagnosis prior to any treatment. They were pathologically confirmed, with 456(91.9%), 31(6.3%) and 9(1.8%) patients be classified as World Health Organization (WHO) type III, II, and I, respectively. Other clinical characteristics were listed in Table 1.

### Treatment

All the included patients received IMRT with or without chemotherapy. A detailed description of the IMRT had been published previously^23^. Of the 472 patients with Stages II–IVB disease, 459(97.2%) were given platinum-based chemotherapy, the sequence used were induction in 71(15.9%), induction-concurrent 139(30.2%), induction-adjuvant 76(16.5%), induction-concurrent-adjuvant 151(32.9%), concurrent 18(3.9%) and adjuvant 2(0.4%), Whenever possible, salvage treatments (including intracavitary brachytherapy, surgery, chemotherapy and boost radiotherapy) were provided for those who developed relapse or persistent disease.

### Single-nucleotide polymorphisms(SNPs) selection and genotyping

Genomic DNA was extracted from whole blood, using the Qiagen DNA Blood Mini Kit (Qiagen, Valencia, CA) according to the manufacturer’s protocol, and stored at −80 **°**C until use. The DNA purity and concentration were determined by spectrophotometric measurement of absorbance at 260 and 280 nm.

Tagging SNPs were selected from 5-kb flanking and within the gene regions of five genes: PIK3CA, AKT1, AKT2, PTEN, and FRAP1 (mTOR) by using the tagger algorithm^24^, and then identified with a cut-off value of r^2^ = 0.8 and a minor allele frequency greater than 0.05 in the Chinese population by Haploview software, based on data from the HapMap Project (www.hapmap.org). Finally, a total of 16 SNPs, including haplotype-tagging SNPs and potential functional SNPs, were selected for genotyping (Table 2).

Among them, 14 SNPs were genotyped by using matrix-assisted laser desorption/ionization-time of flight mass spectrophotometry with the MassARRAY platform (Sequenom, Inc.). Assay data were analyzed using Sequenom TYPER software (version 4.0). Most of the SNPs had a call rate of at least 95%, except two SNPs, with rs2494738 and rs892119 shown to be 93.1% and 94.0%, respectively. Another two SNPs(rs2494732 and rs3803300) were genotyped by TaqMan assay. Randomly repeated assays were used for genotyping quality control.

### Follow-up and statistical analyses

All patients were assessed weekly for treatment response and toxicity during treatment. After the completion of radiotherapy, they were required to be followed-up every three months for the first two years and every 3–6 months during years 3–5. Our study end points were distant metastasis and survival. The overall survival (OS) and distant metastasis-free survival(DMFS) were measured and calculated from the first day of diagnosis to death and distant failure. Data were analyzed using SPSS version 22.0. The Hardy-Weinberg equilibrium of the mutation was determined by *χ*2 test. Univariate and multivariate analyses were performed to define the association between each SNPs and the risk of distant metastasis and death. We also evaluated the combined effects by the number of unfavorable genotypes identified from the main effects analysis of single SNPs. Survival tree analyses were used to derive prognostic groups that combined unfavorable genotypes with N category. Survival tree analysis was performed using the STREE program (http://masal.med.yale.edu/stree/) which uses recursive-partitioning analysis(RPA) to identify subgroups of individuals at higher risk. A two-sided *P*-value of ≤0.05 was considered as statistically significant.

The performance of RPA model was compared with N category and clinical stage, by using Akaike information criterion(AIC)^25^ and Harrell’s concordance index(c-index)^26^. The AIC and c-index were both calculated for the Cox proportional hazards regression model. The AIC refers to the information loss of the selected prognostic model, a smaller AIC value suggests a better goodness of fit of the model. The c-index measures the ability to predict the outcomes, a higher c-index suggests a better discriminatory power of the model. All statistical analyses were conducted with SPSS 22.0 and R3.13.

## Results

### Survival and prognostic analysis

The median follow-up time was 40 months (range 4–51 months) for the whole cohort, with the 3-year OS and DMFS shown to be 91.8% and 85.1%, respectively. At the time of censorship, distant metastasis had developed in 74 patients. The sites of metastases included bone only (n = 21), lung only (n = 14), liver only (n = 20), and other unspecified sites (n = 2), or multiple sites (n = 17). The median time from NPC diagnosis to detection of distant metastasis was 16 months (range 4–47months).

Potential associations between patient-, tumor-, therapy-related characteristics and distant metastasis by univariate and multivariate analyses were tested, as shown in Table 3. We found that N classification was significantly associated with distant metastasis, with patients having advanced N category at higher risk of distant failure (*P* < 0.001). Neither T classification nor treatment factors were associated with distant metastasis in this population. This effect was virtually unchanged after adjustment for gender, age, T classification and cycles of chemotherapy in the Cox model (Table 3).

### Associations between SNPs and survival

Statistical results of χ2 test indicated that the allele frequencies of the enrolled SNPs all fit with Hardy-Weinberg equilibrium (*P* > 0.05, data not shown). Variant genotypes of each of the 16 individual SNP were evaluated for association with DMFS after definitive chemo-radiotherapy, by using univariate and multivariate analysis.

**Figure 1:**
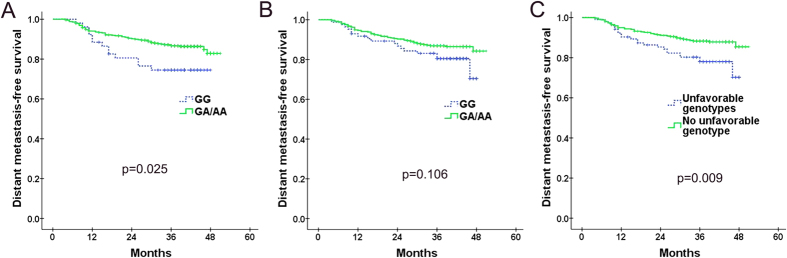
Kaplan-Meier curves of DMFS in NPC patients with different genotypes of (**A**) AKT1: rs3803300 and (**B**) AKT1:rs2494738; (**C**) Kaplan-Meier curves of DMFS in NPC patients with or without unfavorable genotypes.

Of the 16 SNPs, only two SNPs- AKT1: rs3803300 and AKT1:rs2494738-were found to be significantly associated with DMFS after MVA (Table 4). AKT1: rs3803300, as detailed in Table 4, resulted in significantly inferior DMFS for patients with wild-type genotype compared with those with one or two variant alleles (Fig. 1) (74.5% vs. 86.7%, *P* = 0.025). The same result was verified after stratifying by patient-, tumor- and treatment-related factors (HR = 0.536; 95% CI = 0.292–0.986; *P* = 0.045). Patients carrying at least one variant allele for AKT1: rs2494738 had a better DMFS than those with wile-type genotype (86.9% vs. 80.5%, *P* = 0.106) (Fig. 1), this trend became significant after adjusting by gender, age, treatment, T- and N-category (HR = 0.530; 95% CI = 0.302–0.929; *P* = 0.027).

As showed in MVA, another two SNPs-AKT1:rs1130214, and mTOR:rs11121704 showed an obvious trend toward inferior DMFS in patients with one or two variant alleles (AKT1:rs1130214 HR = 1.566, 95% CI = 0.956–2.567, *P* = 0.075; mTOR:rs11121704 HR = 1.655, 95% CI = 0.933–2.935, *P* = 0.085). While AKT2: rs892119 indicated a clear trend to have reduced risk of distant failure when patients carried at least one variant allele (HR = 0.579, 95% CI = 0.317–1.057, *P* = 0.075). Similar analyses of the other 11 SNPs showed no associations between different genotypes and incidence of distant failure (Table 4).

### Combined effect of SNPs on risk of distant metastasis

As these two AKT1 SNPs(AKT1: rs3803300 and AKT1:rs2494738) were consistently associated with risk of distant metastasis, we tried to perform an unfavorable genotype analysis to determine the effect of having one or both of these SNPs. For the 446 patients who had successfully genotyped for these two SNPs, 21, 83 and 342 cases were indicated to carry two, one and none of the unfavorable genotype, respectively. Because of the small number of patients who had two unfavorable genotypes, patients had one or two unfavorable genotype were combined as one group (n = 104), which was indicated to have significant lower DMFS than the other group which had no unfavorable genotype (78.0% vs. 88.3%, *P* = 0.009) (Fig. 1). Multiple Cox proportional hazard analysis with adjustments for T classification, N-classification, gender, age and treatment further confirmed this result (HR = 0.443; 95% CI = 0.264–0.744; *P* = 0.002).

Further subgroup analysis was performed to find out whether the unfavorable genotype had different prognostic role in N0-1 and N2-3 patients. Of the whole group (446 cases), there were 190 patients be classified as N2-3 stage, the MVA analysis indicated that unfavorable genotype was significantly associated with the risk of distant metastasis (HR = 0.456; 95% CI = 0.241–0.863; *P* = 0.016). However, for the 256 patients with N0-1 category, unfavorable genotype seems to had no significant effect on risk of distant failure (HR = 0.058; 95% CI = 0.210–1.228; *P* = 0.133).

### Joint effect of SNPs and other prognostic factors

**Figure 2:**
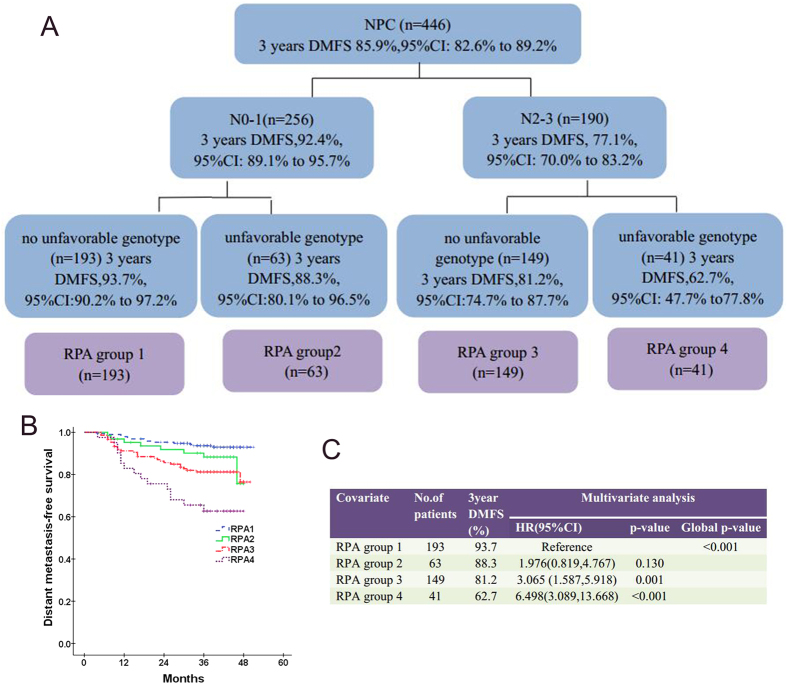
(**A**) Prognostic grouping by recursive-partitioning analysis(RPA) analysis showed the interactions between SNPs and N classification; (**B**) Kaplan-Meier curves of DMFS for four different RPA groups; (**C**) The prognostic effect of RPA grouping on DMFS by multivariate analysis.

In current series, only N classification and unfavorable genotype were identified as factors strongly related to risk of distant failure. RPA for DMFS was conducted with these two factors to derive RPA groups objectively. The RPA algorithm finally classified these patients into four RPA groups: RPA1 (low risk: N0-1, without unfavorable genotype), RPA2 (moderate risk: N0-1, with at least one unfavorable genotype), RPA3 (high risk: N2-3, without unfavorable genotype) and RPA4 (highest risk: N2-3, with at least one unfavorable genotype), with patients in RPA 4 had the highest risk of distant failure (Fig. 2). Multivariate analysis, adjusted for gender, age, T classification and treatment confirmed that higher RPA group conferred an increased distant metastatic risk (HR = 1.783; 95% CI = 1.364–2.331; *P* < 0.001).

In fact, besides RPA model and N category, clinical stage was another significant prognostic factor for DMFS as calculated by MVA, adjusted for gender, age and treatment (HR = 1.438; 95% CI = 1.042–1.982; *P* = 0.027). In order to compare the performance of the RPA model and both N category and clinical stage, the AIC and c-index were introduced in this study. As detailed in Table 5, in comparison with the N category and clinical stage, the RPA model presented with the lowest AIC and the highest c-index. This suggested that the RPA model was the best model in predicting the risk of distant failure, when compared with N category and clinical stage.

## Discussion

There is a growing realization that genetic polymorphisms not only influence the development of cancer, but also the progression of cancer and prognosis^27^. Better understanding of the influence of genetic variations on the clinical outcome of patients may in fact provide additional biomarkers for individualized treatment for NPC. In this study, we determined whether genetic variations in the PI3K/PTEN/AKT/mTOR pathway were associated with risk of distant failure in NPC patients. To our knowledge, this is the first report to apply a tagging SNP approach to evaluating the role of this pathway in clinical outcome for NPC.

We found that SNPs in AKT1: rs3803300 and AKT1: rs2494738 were strongly correlated to risk of distant failure. Patients carried at least one of these two unfavorable genotypes have significant lower DMFS than those had no unfavorable genotype, especially in N2-3 group. Furthermore, we performed RPA for DMFS to derive different prognostic groups that combined SNPs and N classification, which was the only predictor identified to be significant from MVA, especially in patients with N2-3 stage. That was RPA1 (low risk: N0-1, without unfavorable genotype), RPA2 (moderate risk: N0-1, with at least one unfavorable genotype), RPA3 (high risk: N2-3, without unfavorable genotype) and RPA4 (highest risk: N2-3, with at least one unfavorable genotype), with patients in RPA 4 had the highest risk of distant failure. This RPA group was found to be the most optimal model in predicting the risk of distant metastasis, when compared with N category and clinical stage. Our results suggesting that one’s genetic background would be an effective complementary for N classification to evaluate the risk of distant progression.

AKT1, which is the central node of PI3K signaling pathway, has been implicated in the regulation of angiogenesis and metastasis-both important processes in cancer development and progression^28^,^29^. Recent studies have identified that SNPs and their haplotypes of AKT1 were linked with AKT1 protein expression level and with apoptotic capacity^30^,^31^. The most interesting finding of current series was that two SNPs in this gene, AKT1:rs3803300 and AKT: rs2494738 alone or combined, were significantly associated with DMFS in NPC patients. The first SNP AKT1:rs3803300 is located in the 3′ untranslated region of AKT1 and may affect gene expression through changes in transcription factor-binding sites (i.e. RelA and YY1 detected in TRANSFAC programs), microRNA target sequences (i.e. miR-4270 predicted in http://www.bioguo.org/miRNASNP/), and/or splicing variants. Similar results for AKT1: rs3803300 have been observed in other cancers. In a Korea report initiated by Kim MJ *et al*.^15^, the impact of polymorphisms in the AKT1 gene on OS and disease-free survival(DFS) in 310 patients with surgically resected NSCLC were evaluated, and indicated that OS and DFS were significantly higher for patients with the AA genotype of AKT1: rs3803300 than those with GA/GG genotype. This result was then verified by the same Korea group in 814 NSCLC patients with pathologic stages I, II, or IIIA who underwent curative surgical resection^32^. Wang Y *et al*.^8^ from China also indicated that variant genotypes AG and GG of AKT1:rs3803300, especially the GG genotype, showed a strong association with higher Oral squamous cell carcinoma (OSCC) susceptibility than the wild-type genotype AA, but they did not find any association between OSCC progression and genotype distribution of this SNP.

As for AKT1: rs2494738, individuals who carrying at least one variant allele of this SNP had a better DMFS in our analysis. This SNP is located in the intron, when we detected it in http://snpinfo.niehs.nih.gov/, no any related function was found, this may suggested rs2494738 is not the functional variant but a surrogate marker for the underling genetic variation within that region on the genome. Additional studies will be required to identify the causative sequence variation and the mechanism(s) responsible for our observations. However, it is important to note that the AKT1: rs2494738 polymorphism was not found to be associated with prognosis in Caucasian patients with NSCLC and esophageal cancer^11^,^13^. Multiple explanations may underlie this disparity, including ethnicity and different cancer types.

The importance of these two SNPs in determining distant metastasis risk was further supported in a survival tree analysis. We indicated that gene polymorphisms had joint effect with anatomic factor in stratifying patients into different prognostic groups. The present data confirm that N classification is the primary factor that contributing to variation in different risk of distant failure, with this factor demonstrated to be the initial split on the survival tree. We found that the RPA dichotomized the N category into N0-1 versus N2-3. Unfavorable genotype, as has been showed above, permit additional discrimination in the model instead of using N category again and T category. This may implicate that once individuals were defined as N2-3, genetic variation would become the most important factor that influence their ability of distant metastasis. We found that patients in RPA4 (N2-3 with at least one unfavorable genotype) had significantly poorer DMFS rate than that in RPA3 (N2-3 without unfavorable genotype) (62.7% vs. 81.2%), even though it had fewer N3 patients than RPA3(22.0% vs. 32.9%). This observation highlighted the important role of genetic variation in modulating distant metastasis in NPC patients, which may provide additional biomarkers for individualized treatment.

Our results suggest that another SNP rs1130214 in AKT1 may modulate the invasion activity, as patients carrying at least one variant allele of rs1130214 in AKT1 had a clear trend toward higher risk of distant metastasis. This SNP is located in the 5′UTR of AKT1, and has been reported to be associated with the expression of PDK1^33^, which is the phosphoinositide-dependent kinases responsible for phosphorylating AKT, resulting in AKT activation^34^. Similar influence of the SNP in clinical outcome has been reported in other cancers, including NSCLC (Chinese and Koreans), esophageal cancer (90% Caucasian) and prostate cancer (90% Caucasian)^13^,^15^,^35^,^36^. However, another NSCLC study conducted by Xia P *et al*.^11^ from USA observed a conflicting result in non-Hispanic Caucasian patients, they found that AKT1: rs1121304 resulting in significantly decreased risks of distant progression in patients carrying at least one variant allele. Racial difference maybe the primary reason responsible for the variation, different cancer types and sample sizes may contribute to this variation as well.

AKT2 is another pivotal player in the PI3K pathway. The current series found that rs892119 in *AKT2* exhibited significant associations with the hazard of distant metastasis, patients who had at least one variant allele had significant reduction of distant failure. In accordance to our study, Hildebrandt MA *et al*.^13^ indicated that the rs892119 A allele was significantly associated with reduced risk of both recurrence and death, and was also found to be associated with a poorer response to therapy. However, in Wang LE *et al*.’s study^37^, the A allele was found to be linked with increase risk of death in endometrial cancer in Caucasian population.

mTOR is another critical regulator of the PI3K pathway, which was reported to be involved in several cellular processes, including carcinogenesis, proliferation, angiogenesis and metabolism^38^. The current study found that patients with the CT/CC genotype of mTOR: rs11121704 tended to have higher risk of distant failure than those with TT genotype. However, an American study conducted in Caucasians indicated that the TT genotype of rs11121704 was associated with poor survival and resistance to chemotherapy^13^. Ethnicity difference was considered as the main reason for this phenomenon, since this study exhibited an entirely different genotype distribution, higher proportion of TT genotype was noticed in our series based on Chinese Han population (85.1%) than the American study mainly based on Caucasian (8.2%). Different sample sizes and cancer types may be another issues that resulted in variant outcomes.

Several limitations should be addressed in our series. First of all, the retrospective nature of the current study certainly served as an inherited and fundamental pitfall. Secondly, although the five genes included in this analysis were the core functional components of the pathway, this pathway is complex, with several other genes warranting investigation, such as PDK1, PDK2, TSC1, and TSC2, as have been mentioned in other literatures^13^,^14^,^16^. This may contribute to additional variation in clinical outcome, especially in combination with genetically altered AKT. Moreover, our analysis was based on single institution and only in Chinese Han population, additional studies in other ethnicities and institutions are required to confirm our findings. Lastly, since the variants genotyped in this study were tagging SNPs, we are unable to identify all the causative SNP and mechanism responsible, future studies are clearly warranted in this regard.

In conclusion, polymorphisms in the PI3K/PTEN/AKT/mTOR pathway were found to be independent prognostic markers for NPC patients, especially in N2-3 patients. Consequently, in addition to anatomic factors, testing for the presence of these polymorphisms may help with identifying patient subgroups at high risk of distant failure. If the findings from the current study could be validated prospectively in multicenter and diverse ethnic populations, these results, in combination with clinical-pathologic data, could become the basis for selecting patient subgroups at high risk of distant metastasis, thereby helping to refine therapeutic decisions in the treatment of NPC.

## Additional Information

**How to cite this article**: Guo, Q. *et al*. Genetic variations in the PI3K-PTEN-AKT-mTOR pathway are associated with distant metastasis in nasopharyngeal carcinoma patients treated with intensity-modulated radiation therapy. *Sci. Rep.* **6**, 37576; doi: 10.1038/srep37576 (2016).

**Publisher's note:** Springer Nature remains neutral with regard to jurisdictional claims in published maps and institutional affiliations.

## Figures and Tables

**Table 1:** Patients’ characteristics.

Covariate	n = 496(%)
Alive or death	
alive	451 (90.9)
death	45 (9.1)
Distant Metastasis	
no	422 (85.1)
yes	74 (14.9)
Gender	
male	321 (64.7)
female	175 (35.3)
Age (years)	
≤45	230 (46.4)
>45	266 (53.6)
T classification	
T1	126 (25.4)
T2	81 (16.3)
T3	118 (23.8)
T4	171 (34.5)
N classification	
N0	51 (10.3)
N1	234 (47.2)
N2	147 (29.6)
N3	64 (12.9)
Clinical Stage	
Stage I	24 (4.8)
Stage II	101 (20.4)
Stage III	159 (32.1)
Stage IV	212 (47.2)
Treatment Modality	
chemoradiotherapy	467 (94.2)
radiotherapy alone	29 (5.8)
Total Cycles Of Chemotherapy	
≤3	188 (37.9)
>3	308 (62.1)

**Table 2:** Tagging SNP Characteristics.

SNP	Alleles	SNP type	SNP location	Detectable rate
PI3KCA				
rs6443264	T/G	intron variant	chr3:9781618	100%
rs2699887	G/A	intron variant	chr 3:179148620	99.0%
AKT1				
rs1130214	G/T	utr variant 5 prime	chr14:104793397	96.7%
rs3803304	G/C	intron variant	chr 14:104772809	100%
rs2494738	G/A	intron variant	chr 14:104780349	93.1%
rs2498804	T/G	upstream variant 2 kb	chr 14:104766758	99.0%
rs2494732	T/C	intron variant	chr 14:104772855	96.2%
rs3803300	G/A	utr variant 3 prime	chr 14:104803442	96.0%
AKT2				
rs2304186	G/T	utr variant 3 prime	chr 19:40233814	98.4%
rs892119	G/A	intron variant	chr 19:40254165	94.0%
mTOR				
rs11121704	T/C	intron variant	chr 1:11233902	100%
rs2295080	G/T	upstream variant 2 kb	chr 1:11262571	100%
PTEN				
rs2299939	C/A	intron variant	chr 10:87897393	99.6%
rs11202607	C/T	utr variant 3 prime	chr 10:87967657	100%
rs701848	T/C	utr variant 3 prime	chr 10:87966988	99.0%
rs12569998	T/G	intron variant	chr 10:87914400	99.8%

**Table 3:** Prognostic analysis of clinical characteristics for DMFS.

Covariate	Univariate analysis		Multivariate analysis	
	HR (95%CI)	p-value	HR (95%CI)	p-value
**age**	1.007 (0.987, 1.028)	**0.472**	1.008 (0.988, 1.029)	**0.410**
**gender**		**0.527**		**0.578**
male	reference		reference	
Female	0.853 (0.521,1.396)		0.869 (0.530,1.425)	
**T classification**		**0.915**		**0.950**
T1	reference		reference	
T2	1.082(0.515,2.267)		1.006 (0.479,2.116)	
T3	1.062 (0.542,2.080)		0.987 (0.500,1.945)	
T4	1.229 (0.673,2.246)		1.149 (0.625,2.113)	
**N classification**		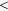 **0.001**		**<0.001**
N0	reference		reference	
N1	2.322 (0.544,9.902)		2.509 (0.580, 10.853)	
N2	5.899 (1.412,24.650)		6.507 (1.526, 27.740)	
N3	9.350 (2185,40.019)		10.807 (2.451,47.653)	
**Chemotherapy-**		**0.634**		**0.418**
≤3 cycles	reference		reference	
>3 cycles	1.123 (0.697,1.810)		0.808 (0.483,1.353)	

**Table 4:** Prognostic analysis of individual SNPs for DMFS.

Covariate	No. of patients	Univariate analysis		Multivariate analysis	
		3y DMFS(%)	p-value	HR (95%CI)	p-value
**PI3KCA:rs6443264**			0.069		0.151
TT	68	92.0		Reference	
TG or GG	428	84.2		1.958 (0.782,4.905)	
**PI3KCA:rs2699887**			0.384		0.456
GG	451	84.7		Reference	
GA or AA	40	89.7		0.680 (0.247,1.875)	
**AKT1:rs1130214**			0.029		**0.075**
GG	369	86.9		Reference	
GT or TT	111	78.0		1.566 (0.956,2.567)	
**AKT1:rs3803304**			0.592		0.649
CC	412	84.7		Reference	
CG or GG	84	86.8		0.861(0.452,1.640)	
**AKT1:rs2494738**			0.106		**0.027**
GG	85	80.5		Reference	
GA or AA	377	86.9		0.530 (0.302,0.929)	
**AKT1:rs2498804**			0.208		0.221
TT	203	87.6		Reference	
TG or GG	288	83.2		1.354 (0.833,2.200)	
**AKT1:rs249732**			0.162		0.124
CC	257	87.6		Reference	
CT or TT	220	83.1		1.450 (0.903,2.328)	
**AKT1:rs3803300**			0.025		**0.045**
GG	52	74.5		Reference	.
GA or AA	424	86.7		0.536 (0.292,0.986)	
**AKT2:rs2304186**			0.757		0.625
GG	115	88.0		Reference	
GT or TT	373	84.6		1.151(0.656,2.020)	
**AKT2:rs892119**			0.219		**0.075**
GG	356	84.1		Reference	
GA/AA	110	88.0		0.579 (0.317–1.057)	
**mTOR:rs11121704**			0.165		**0.085**
TT	422	86.3		Reference	
CT or CC	74	79.5		1.655 (0.933,2.935)	
**mTOR:rs2295080**			0.949	0	0.739
TT	307	85.6		Reference	
GT or GG	189	84.7		1.065 (0.665,1.707)	
**PTEN:rs2299939**			0.470		0.560
CC	331	84.5		Reference	
CA or AA	163	86.7		0.862 (0.522,1.422)	
**PTEN:rs11202607**			0.460		0.569
CC	385	84.4		Reference	
CT or TT	111	88.1		0.844 (0.471,1.512)	
**PTEN:rs701848**			0.660		0.488
TT	162	84.7		Reference	
TC or CC	329	85.3		0.844 (0.523,1.363)	
**PTEN:rs12569998**			0.539		0.458
TT	198	83.4		Reference	
TG/GG	297	86.5		0.840 (0.529–1.333)	

**Table 5:** Comparison of risk groups based on RPA model, N category and clinical stage.

	RPA model	N category	Clinical stage
Patients distributions	RPA1:43.3%	N0:10.5%	Stage I:5.2%
	RPA2:14.1%	N1:46.9%	Stage II:20.2%
	RPA3:33.4%	N2:29.6%	Stage III:31.8%
	RPA4:9.2%	N3:13.0%	Stage IV:42.8%
5y-DMFS(%)	RPA1:93.7%	N0:95.5%	Stage I:100%
	RPA2:88.3%	N1:91.7%	Stage II:88.6%
	RPA3:81.2%	N2:79.8%	Stage III:86.1%
	RPA4:62.7%	N3:70.6%	Stage IV:82.4%
P value	p < 0.001	p < 0.001	0.027
AIC	741.252	745.145	759.345
C-index	0.795	0.793	0.762
